# RealtimeDecoder: A Fast Software Module for Online Clusterless Decoding

**DOI:** 10.1523/ENEURO.0252-24.2025

**Published:** 2025-12-16

**Authors:** Joshua P. Chu, Michael E. Coulter, Eric L. Denovellis, Trevor Thai K. Nguyen, Daniel F. Liu, Xinyi Deng, Uri T. Eden, Caleb T. Kemere, Loren M. Frank

**Affiliations:** ^1^ Department of Electrical and Computer Engineering, Rice University, Houston, Texas 77005; ^2^Department of Physiology, University of California, San Francisco, California 94158; ^3^Kavli Institute for Fundamental Neuroscience, University of California, San Francisco, California 94158; ^4^Howard Hughes Medical Institute, University of California, San Francisco, California 94158; ^5^ SpikeGadgets, Inc., San Francisco, California 94158; ^6^ Department of Statistics and Data Science, Beijing University of Technology, Beijing 100021, China; ^7^ Department of Mathematics and Statistics, Boston University, Boston, Massachusetts 02215

**Keywords:** brain–machine interface, hippocampus, learning, memory, real-time neural decoding

## Abstract

Decoding algorithms provide a powerful tool for understanding the firing patterns that underlie cognitive processes such as motor control, learning, and recall. When implemented in the context of a real-time system, decoders also make it possible to deliver feedback based on the representational content of ongoing neural activity. That, in turn, allows experimenters to test hypotheses about the role of that content in driving downstream activity patterns and behaviors. While multiple real-time systems have been developed, they are typically implemented with a compiled programming language, making them more difficult for users to quickly adapt for new experiments. Here we present a software system written in the widely used Python programming language to facilitate rapid experimentation. Our solution implements the state space based clusterless decoding algorithm for an online, real-time environment. The parallelized application processes neural data with temporal resolution of 6 ms and median computational latency <50 ms for medium- to large-scale (32+ tetrodes) rodent hippocampus recordings without the need for spike sorting. It also executes auxiliary functions such as detecting sharp wave ripples from local field potential data. Even with an interpreted language, the performance is similar to state-of-the-art solutions that use compiled programming languages. We demonstrate this real-time decoder in a rat behavior experiment in which the decoder allowed closed-loop neurofeedback based on decoded hippocampal spatial representations. Overall this system provides a powerful and easy-to-modify tool for real-time feedback experiments.

## Significance Statement

We developed a pure Python software system to decode hippocampal spiking activity in real-time without the need for spike sorting. Our solution was validated in a real-time closed-loop experiment and can be customized for different data acquisition systems. This represents a valuable new resource to enable experiments requiring low-latency, closed-loop interaction with the information encoded in neural ensembles.

## Introduction

The brain enables animals to keep track of information about internal states and the external world and to use that information to guide action selection. This tracking engages neural representations, and thus understanding how those representations relate to internal or external variables can help us understand mental processes (Knierim, 2014). Decoding analyses provide one approach to understanding neural representations: an initial encoding model is built that relates observed variables to spiking, and then this model is inverted to enable predictions of observed variables based on spiking data (Brown et al., 1998). This approach has been used to characterize representations of neural activity from brain regions such as the hippocampus (Davidson et al., 2009; Karlsson and Frank, 2009; Pfeiffer and Foster, 2013).

The classic application of decoding was to assess how well a given variable could be read out from ongoing neural population activity when that variable (e.g., the position of a limb or of the animal in space) could be observed. When such a correspondence has been established, decoding can also provide insight into representations expressed in the absence of an observable variable. In the hippocampus, for example, the spiking of “place cells” can be decoded both during movement and during periods of immobility. Strikingly, there are times when this spiking corresponds not to current location but instead to other places in the animals environment or even other environments (Carr et al., 2011; Foster, 2017; Ólafsdóttir et al., 2018; Pfeiffer, 2020). Similarly, decoding has also enabled the development of neurofeedback systems, such as brain–machine interfaces, that can translate neural activity patterns into useful outputs (e.g., moving a cursor on a screen or generating speech in patients with paralysis; Daly and Wolpaw, 2008; Luo et al., 2023).

Historically, decoding hippocampal spatial activity patterns used a decoder which relied on sorted spikes (spikes that can be “clustered” and thereby assigned with reasonable confidence to a single neuron; Diba and Buzsáki, 2007; Davidson et al., 2009; Karlsson and Frank, 2009; Gupta et al., 2010; Pfeiffer and Foster, 2013; Grosmark and Buzsáki, 2016; Wu et al., 2017; Davoudi and Foster, 2019; Farooq et al., 2019; Shin et al., 2019; Zheng et al., 2021). In this decoder, a Poisson model was used to describe the neural dynamics of individual place cells, where the Poisson rate is directly related to the place field (Brown et al., 1998; Zhang et al., 1998; van der Meer et al., 2017).

One disadvantage of the sorted spikes decoder is that it excludes lower amplitude or otherwise non-clusterable spikes. These spikes nevertheless contain valuable information for decoding, and alternative models known as clusterless decoders have been developed (Kloosterman et al., 2014; Deng et al., 2015, 2016; Williams et al., 2020; Denovellis et al., 2021). These decoders use many more of the recorded spikes (typically all that pass a specific amplitude threshold) and provide more accurate decoding compared to sorted spike decoders in cases that have been tested (Kloosterman et al., 2014; Deng et al., 2015). Subsequent studies have applied these methods to derive scientific conclusions (Hu et al., 2018; Michon et al., 2019; Gillespie et al., 2021).

Clusterless decoding thus offers a powerful tool for identifying the content of neural representations, and real-time implementations have the potential to enable the use of this tool in closed-loop experiments. Current implementations are written in a compiled programming language, however, which increases the difficulty of customization for end users without more advanced programming experience (Ciliberti et al., 2018). We therefore developed RealtimeDecoder our software program that implements the state space models in Denovellis et al. (2021) for online, real-time clusterless decoding. The system is parallelized and written entirely in Python for ease of use for both users and developers. Despite using an interpreted language, the software achieves computational latencies similar to state-of-the-art solutions. An added benefit is the implementation of a state space model, allowing the experimenter to use both likelihood and posterior for downstream analysis.

In this work, we describe the architecture and performance of RealtimeDecoder. We focus on the latencies of computations and feedback, which are especially relevant in a real-time context. We also demonstrate the system’s use in a live closed-loop experiment for proof of concept, and we briefly discuss some results. Our hope is that this work will help advance scientific research by enabling other closed-loop experiments that can elucidate the role of hippocampal spatial representations.

## Materials and Methods

### Code accessibility

The code/software described in the paper is freely available online at https://github.com/LorenFranklab/realtime_decoder. The code is available as Extended Data. All results were obtained on two Ubuntu 20.04 desktops equipped with 32-core processors (AMD 3970x Threadripper or AMD Epyc 7452).

10.1523/ENEURO.0252-24.2025.d1 Data 1 This file contains the python scripts necessary to run the RealtimeDecoder software. These scripts run all processes described above. Also included in this zip file are configuration files used to customize the software for each application. The most up-to-date version of the code can be found at: https://github.com/LorenFrankLab/realtime decoder. Download Data 1, ZIP file.

10.1523/ENEURO.0252-24.2025.d2 Data 2 This file contains the python scripts to run the RealtimeDecoder software as well as sample raw data files (neural data and camera data) that allow the user to demonstrate the functionality of the software. Download Data 2, ZIP file.

### Model formulation

The clusterless decoder used in this work is based on Deng et al. (2015) and Denovellis et al. (2021). Similar to a sorted spikes decoder, this decoder uses a recursive Bayesian framework. The output at each timestep is an estimate of the distribution of the decoded position that combines information from the previous timestep with the spiking data observed during the current timestep:

$$p(x_{k}|\Delta N_{k},\{{\vec{m}}_{k}\})=\frac{\;p(x_{k})\;p(\Delta N_{k},\{{\vec{m}}_{k}\}\;|\;x_{k})}{p(\Delta N_{k},\{{\vec{m}}_{k}\})}.$$

In this equation *x*_*k*_ is decoded position for time bin *k*, Δ*N*_*k*_ is the number of spikes emitted, and $\left\{{\vec{m}}_{k}\right\}$ is the set of marks of length Δ*N*_*k*_, that is, vectors associated with each spike observed in time bin *k*. In practice, the marks used in the model are spike waveform features such as peak amplitudes.

The distribution *p*(*x*_*k*_) on the right side of Equation 1 is given by:

$$p(x_{k})=\int p(x_{k}\;|\;x_{k-1})\;p(x_{k-1}\;|\;\Delta N_{k-1},{\vec{m}}_{k-1})dx_{k-1}.$$

This causal relationship updates the estimate of the animal’s positions across time based on the decoder output from the previous time step using a model of the animal’s movement across time, *p*(*x*_*k*_ | *x*_*k*−1_). Here we assumed that (Eq. 1) transitions from *x*_*k*−1_ to *x*_*k*_ are Markovian (Deng et al., 2015).

For the clusterless likelihood, the probabilistic model relating observed data and animal position assumes that each spike contributes equally, and is thus a product across electrodes and across spikes within each electrode of the Poisson likelihood of observing the mark that was recorded. Mathematically, this is:

$$\begin{array}{rl} \;p(\Delta N_{k},{\vec{m}}_{k}\;|\;x_{k}) & =p(\Delta N_{k}^{\left(1\;:\;E\right)},{\left\{{\vec{m}}_{k,j}^{i}\right\}}_{\;j=1\;:\;\Delta N_{k}^{i}}^{i=1\;:\;E}\;|\;x_{k}),\\ & \propto \prod_{i=1}^{E}\prod_{\;j=1}^{\Delta N_{k}^{i}}[\lambda_{i}(t_{k},{\vec{m}}_{k,j}^{i}\;|\;x_{k})\Delta_{k}]\;\text{exp}[-\Lambda_{i}(t_{k}\;|\;x_{k})\Delta_{k}], \end{array}$$

where *t*_*k*_ is the time bin *k*, Δ_*k*_ is the time bin size, and $\prod_{\;j=1}^{\Delta N_{k}^{i}}[\lambda_{i}(t_{k},{\vec{m}}_{k,j}^{i}\;|\;x_{k})\Delta_{k}]\text{\textbackslash{} }exp[-\Lambda_{i}(t_{k}\;|\;x_{k})\Delta_{k}]$ is the likelihood for electrode group *i* = 1, …, *E*. For each electrode group, the product $\left[\lambda_{i}(t_{k},{\vec{m}}_{k,j}^{i}\;|\;x_{k})\Delta_{k}]\text{\textbackslash{} }exp[-\Lambda_{i}(t_{k}\;|\;x_{k})\Delta_{k}\right]$ is computed for every spike *j* within time bin *k*. If there are no spikes observed for electrode group *i* in time bin *k*, then that electrode group’s likelihood becomes:

$$\exp\;[-\Lambda_{i}(t_{k}\;|\;x_{k})\Delta_{k}],\;\Delta N_{k}^{i}=0.$$

The likelihood consists of the intensity functions:

$$\lambda_{i}(t_{k},{\vec{m}}_{k,j}^{i}\;|\;x_{k})=\mu_{i}\frac{\;p_{i}(x_{k},{\vec{m}}_{k,j}^{i})}{\pi (x)},$$

$$\Lambda_{i}(t_{k}\;|\;x_{k})=\mu_{i}\frac{\;p_{i}(x_{k})}{\pi (x)},$$

where Equation 5 is the joint mark intensity (JMI) and Equation 6 is the ground process intensity. The JMI is a function that specifies the rate of observing a spike with a particular mark at a given position. The ground process intensity is a function of the overall spike rate at a given position, calculated by marginalizing out the individual marks, and accounts for the absence of spikes at a given time bin. *μ*_*i*_ is the mean firing rate of electrode group *i* and *π*(*x*) is the occupancy, a distribution of positions the animal has visited. *p*_*i*_(*x*_*k*_) is the probability of observing any spike given for electrode *i*, given the position. Lastly $p_{i}(x_{k},{\vec{m}}_{k,j}^{i})$ represents a joint probability distribution over position and marks. During the model learning phase, no computation is performed–each mark $\vec{m}$ is simply saved along with the position *x* at which the mark was observed. Once the learning phase is complete, $p(x,\vec{m})$ is estimated on-the-fly for each observed mark ${\vec{m}}_{l}$. First a weight is computed for each mark ${\vec{m}}_{o}$ that has been saved in the model, using a Gaussian kernel:

$${a_{o}}^{2}=\sum_{d=1}^{D}{\left({\vec{m}}_{o,d}-{\vec{m}}_{l,d}\right)}^{2},$$

$$w_{o}=\frac{1}{\sigma \sqrt{2\pi }}\exp\;\left[-\frac{{a_{o}}^{2}}{2\sigma^{2}}\right],$$

where *D* is the number of mark dimensions and *σ* is a user-defined parameter. Then the estimate $p(x,\vec{m})$ evaluated at the mark ${\vec{m}}_{l}$ is computed by taking a weighted histogram using the positions {*x*_*o*_} saved during the learning phase and the corresponding weights {*w*_*o*_} described above.

Finally the posterior distribution (i.e., the recursive update at time step *k*) can be written as:

$$p(x_{k}\;|\;\Delta N_{k},\{{\vec{m}}_{k}\})\propto p(x_{k})\;p(\Delta N_{k},\{{\vec{m}}_{k}\}\;|\;x_{k}),$$

where *p*(*x*_*k*_) is given in Equation 2 and $p(\Delta N_{k},\{{\vec{m}}_{k}\}\;|\;x_{k})$ is given in Equation 3.

### Software architecture

Since the clusterless likelihood in Equation 3 is a product over electrode groups, this formula presents a straightforward scheme for parallelization. Each factor in can be computed simultaneously before being combined into the overall likelihood. The likelihood is then used to estimate the posterior distribution in Equation 9.

**Figure 1.**
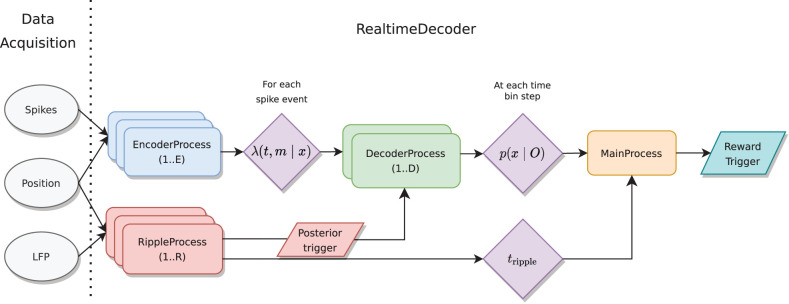
Data flows through different Process nodes in the RealtimeDecoder system, which are responsible for different computations. O represents $\Delta N,\{\vec{m}\},H$ for a given time bin. Note that in most cases, one DecoderProcess is sufficient, but the system can by default support two DecoderProcesses in the MainProcess’s logic that triggers a reward.

To carry out these computations RealtimeDecoder uses three different input data streams: local field potential (LFP), spike events (particularly the waveform data), and position. An overview of the computational approach is shown in Figure 1. Input data (ellipses) flow to parallel processes (rounded rectangles) which compute intermediate quantities (rhomboids), resulting in a reward trigger output (parallelogram) if a specific, experimenter specified representation is detected. To send messages between different processes, we used the low-latency messaging protocol message passing interface (MPI;Walker and Dongarra, 1996).

The primary objective of online real-time clusterless decoding is to estimate the posterior distribution (Eq. 9), which is a conditional probability distribution over a variable *x*. In this application, *x* represents linearized (1D) position. Recall that the clusterless likelihood in Equation 3 is a product over electrode groups, which presents an obvious target over which to parallelize. The overall software architecture reflects this feature, as the computation of Equation 3 can be split among multiple processes to reduce total system latency. These sub-computations can then be aggregated to form the posterior probability distribution.

Additionally, the system can process LFP data to extract time boundaries in which LFP events (specifically sharp wave ripples or SWRs) occur. Experimenters thus have multiple options when using the system; for example, they may specify that only replay with a concomitant SWR will trigger neurofeedback.

RealtimeDecoder is implemented as different Process objects, namely MainProcess, RippleProcess, EncoderProcess, and DecoderProcess which serve different computational functions (Fig. 1). Each instance of these objects (with the exception of the event-driven GuiProcess) implements a polling while loop which typically consists of the pseudocode in Box 1:

Box 1. Pseudocode example for a Process object

**while** no errors or stop signal not triggered **do**

 check for messages from other processes

 **if** message ready

  process message

 **endif**

 **if** data ready

  process data

  sent results to other processes

  write results

 **endif**

**endwhile**

#### MainProcess

The MainProcess coordinates all other Process instances by gathering information about the binary data they output to file and monitoring their status to check for runtime errors, among other functions. Inside the MainProcess runs a custom data handler that processes data computed by other Process objects, such as position information, ripple onset times, a new posterior distribution estimate, etc. In our usage, this object detects replay events; upon detection, it sends a signal to dispense reward.

#### RippleProcess

An instance of a RippleProcess processes LFP data and is primarily responsible for detecting SWRs. This occurs via the following procedure: (1) LFP from the data acquisition system is band-pass filtered to the SWR frequency band (150–250 Hz), (2) an estimate of SWR power is obtained by applying a low pass filter to the square of the band-pass filtered data, and finally (3) the baseline (mean) power is estimated. The start of a SWR is then marked when the z-score of this power estimate exceeds a user-defined threshold (typically at least 3).

#### EncoderProcess

An instance of an EncoderProcess manages the encoding model for one or more electrode groups (e.g., a single tetrode). Typically if the number of available threads permits, a single instance will handle just one electrode group for minimum computational delay. During the training period, an EncoderProcess adds spikes to the encoding model and also computes an estimate of the JMI (Deng et al., 2015). When the training period is over, spikes are no longer added to the encoding model, but the JMI continues to be estimated.

#### DecoderProcess

The DecoderProcess gathers the JMI functions sent by instances of an EncoderProcess. At every user-defined time bin step, it updates the likelihood and posterior distribution of the clusterless decoding state space model. This update is triggered by exactly one RippleProcess which keeps time based on the timestamped LFP data it receives. A DecoderProcess sends these estimates to the MainProcess to be further processed by a custom data handler. This data handler is developed according to the needs of the particular experiment and may implement features such as remote spatial representation detection.

#### GuiProcess

The GuiProcess increases user-friendliness of the system. It consists of a visualization window that displays the likelihood, posterior, and state probabilities in real-time. It also includes a dialog window that allows the user to change parameters during the course of the experiment, as well as some control options to start and stop the software as a whole.

#### System characterization

**Figure 2.**
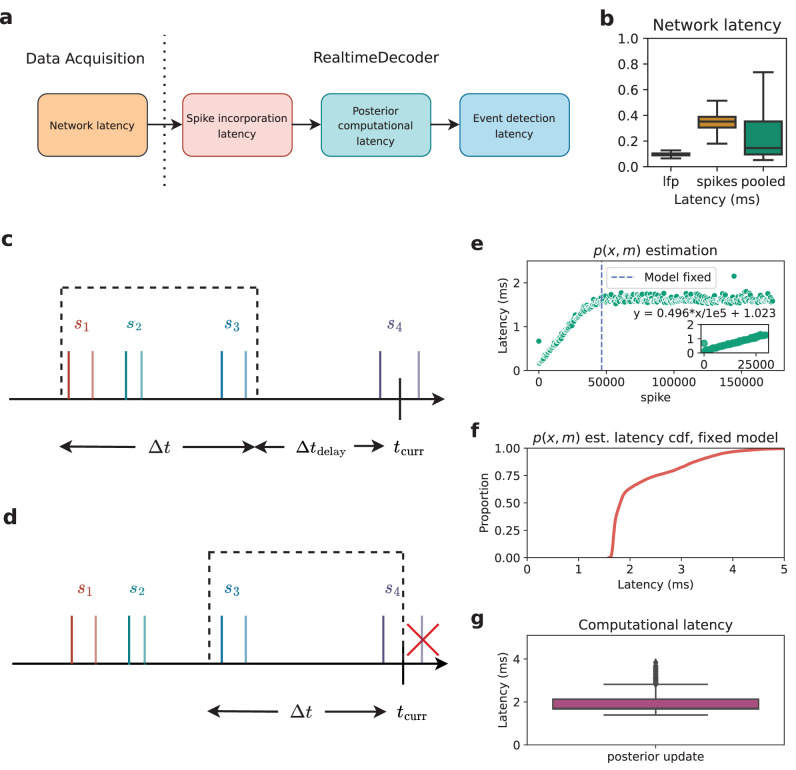
***a***, The four major components contributing to overall latency. ***b***, Network latency. ***c–d***, Four spikes are shown. The darker shade indicates when a spike occur, and the lighter shade indicates when it is in usable form for a DecoderProcess. Note that spike 4 is in usable form at a time later than *t*_curr_. ***c***, Time bin for when Δ*t*_delay_ > 0. ***d***, Time bin for when Δ*t*_delay_ = 0. ***e***, *p*(*x*, *m*) estimation latencies as a function of number of spikes processed by an EncoderProcess. Note that data have been sampled to reduce the size of this figure. ***f***, Cumulative distribution of *p*(*x*, *m*) estimation latencies once the model is fixed. ***g***, Computational latency induced by update of the posterior distribution.

The utility of an online decoding system for real-time feedback depends in large part on its latency. For example, if one chooses too small of a time step to update the posterior distribution estimate, the system cannot compute the necessary quantities quickly enough to be suitable for real-time use. The overall latency stems from the latencies of four major components (Fig. 2*a*): (1) Network latency, (2) spike incorporation latency, (3) posterior computational latency, and (4) event detection latency, which are further expanded in panel (b). Each of these latencies is further explained in subsequent sections.

All latencies were measured using an AMD 3970x Threadripper workstation running the Ubuntu 20.04 operating system. The workstation ran the Trodes data acquisition software in playback mode. In this mode the raw neural data were streamed from a file at a desired sampling rate of 30 kHz. DecoderProcess received data from playback-mode Trodes over the localhost network interface.

#### Network latency

The network latency (Fig. 2*b*) is the time difference between when data (spikes, LFP, position) is sent out by the data acquisition system, and when that data is actually requested by RealtimeDecoder. This type of latency depends on different factors such as network speed, the type of machine running the decoder, etc. Spikes have a higher latency than LFP due to its size, as the entire spike waveform/snippet is transmitted over the network. Nevertheless the median network latency of the pooled data (spikes and LFP combined) is <1 ms when the data acquisition program executes on the same host machine as RealtimeDecoder.

#### Spike incorporation latency

The spike incorporation latency refers to the maximum length of time between when a spike occurs and when it is sent to the decoder in a usable form. To be usable the JMI must be estimated with the spike and be visible to a DecoderProcess instance. At that point in time, the spike is ready to be incorporated into the posterior distribution estimation via an update of the likelihood.

The spike incorporation latency is a user-defined parameter and therefore a constant value. It must be long enough to account for the latency incurred from estimating the JMI, a step whose computation time typically increases with the number of spikes used to generate the encoding models utilized by the decoder.

In RealtimeDecoder, the estimation of the posterior distribution occurs one time bin at a time. The data incorporated into each estimation step are those contained within a certain time window. This window is directly impacted by two user-defined parameters Δ*t*_delay_ and Δ*t*, which are explained below.

*t*_curr_ refers to the time at which an update to the posterior occurs. Δ*t* is the time bin size. Δ*t*_delay_ (a non-negative value) refers to how far behind *t*_curr_ should define the upper edge of the window. A combination of Δ*t*_delay_ and Δ*t* defines the lower edge. In other words, the window is defined by $\left(t_{\mathrm{curr}}-\Delta t_{\mathrm{delay}}-\Delta t,\;t_{\mathrm{curr}}-\Delta t_{\mathrm{delay}}\right)$. The spike incorporation latency is the sum Δ*t*_delay_ + Δ*t*.

Figure 2*b,c* illustrates the effect of the spike incorporation latency on the boundaries of the time window when an update to the posterior is requested at time *t*_curr_. In Figure 2*c*, Δ*t*_delay_ is chosen to be a non-zero value so the upper edge of the window lies at a time prior to *t*_curr_. Spikes *s*_1_, *s*_2_, and *s*_3_ are incorporated into the update of the posterior.

If Δ*t*_delay_ = 0 (Fig. 2*d*), then the upper edge of the window is simply *t*_curr_. However, spike *s*_4_ is NOT incorporated into the posterior update because it has not been transformed into a usable form until a time later than *t*_curr_. For this reason, it is advised to set Δ*t*_delay_ > 0.

Figure 2*e,f* illustrates some considerations for determining an appropriate value to set as the spike incorporation latency. The JMI in Equation 5 must be estimated, where estimation of *p*(*x*, *m*) is the most computationally expensive component. Figure 2*e* shows that the estimation latency increases as more spikes are added to the encoding model. When the model is considered trained, it is fixed. Thus spikes are still processed, but they are not added to the model, and the estimation latency no longer increases linearly. On our test machine, the estimation latency increased at an approximate rate of 0.5 ms additional latency for every 10,000 additional spikes in the encoding model. Figure 2*f* shows the cumulative distribution of the *p*(*x*, *m*) estimation latency once the model in Figure 2*e* is fixed.

In general, the more spikes are expected to be added to the encoding models, the higher the user must set the value of the spike incorporation latency. Too low of a value would mean some JMI’s cannot be computed and incorporated into the posterior estimation step in time, which could adversely affect the quality and accuracy of the posterior.

#### Posterior computational latency

The posterior computational latency is the computation time required to estimate the posterior distribution for a single update step. Figure 2*g* shows the distribution of this latency (median value 1.722 ms on our test machine). The latency is affected by multiple factors, including how many electrodes RealtimeDecoder is configured to analyze and how many states are in the state space model. The distribution in Figure 2*g* is useful for informing the user what the time bin size Δ*t* should be. We advise setting Δ*t* to at least the 75th percentile of this distribution. If Δ*t* is too small, then the decoder would not be able to estimate the posterior quickly enough and would fail to operate in real-time.

#### Event detection latency

Similar to the spike incorporation latency, the event detection latency is a constant value. It is a user-defined parameter that specifies how much of the most recently decoded activity should be used for event detection. Event detection is given by the following procedure: suppose *t*_curr_ is the time at which an update to the posterior occurs. Subsequently a window is drawn with bounds [*t*_curr_ − Δ*t*_event_, *t*_curr_], where Δ*t*_event_ is the event detection latency. Within this window, custom logic is applied to determine whether an event has occurred.

The event detection latency is intended to reduce the number of spurious detections by looking at decoded activity prior to the current decoded activity. A higher value of the event detection latency will theoretically reduce the number of spurious detections, at the cost of increasing the reaction time to a true event.

#### Summary

Overall our decoder performs comparably to state-of-the-art solutions (Table 1) despite being written entirely in Python.

**Table 1.** RealtimeDecoder latency performance relative to the decoder in Ciliberti et al. (2018)

Decoder	Network latency	Spike incorporation latency	Posterior computation latency	Event detection latency
Ciliberti 2018 (32 tetrodes)	0 ms (median, localhost)	10 ms	∼2.2 ms (median)	30 ms
This work (49 tetrodes)	0.102 ms (median, localhost)	12 ms	1.722 ms (median)	30 ms

### Scalability

Box 2. Test input generation for characterizing effect of mark dimensionality on computational latency.

import numpy as np

def generate_mark_data(mark_dim, num_marks_in_model, max_position_bin):

  marks = np.ones((num_marks_in_model, mark_dim))

  positions = np.zeros(num_marks_in_model)

  for ii in range(num_marks_in_model):

    positions[ii] = ii % max_position_bin

  return marks, positions

**Figure 3.**
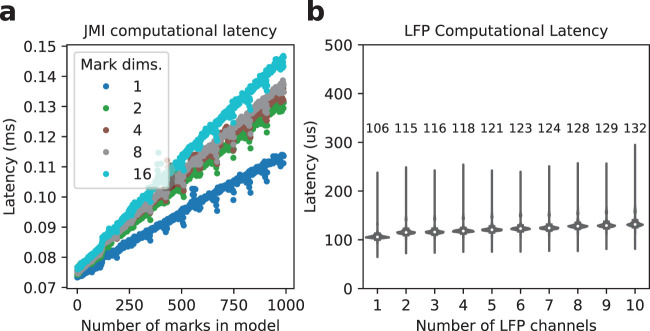
***a***, Computational latency for estimation of the JMI depends on the mark dimensionality. ***b***, LFP computational latency. Median values are labeled above the distribution they were computed from.

The estimation of the JMI depends on the mark dimensionality, where Figure 3*a* demonstrates this relation. Here test inputs were generated deterministically according to Box 2, where all spikes were identical in magnitude and the distribution of positions was uniform. Note that different test inputs are not expected to change the relations demonstrated by Figure 3*a* since the actual values of the marks and position do not matter when only latency measurements are of concern.

Although the computational latencies increase with the number of mark dimensions, the multiplier for this increase is less than the ratio of a given mark dimensionality to a reference mark dimensionality. As an example, increasing the dimensionality from 1 to 16 does not result in a 16X increase in the computational latencies. This is a favorable characteristic especially for experimenters using larger electrode groupings, such as for polymer or silicon multielectrode arrays.

For the LFP processing path, users may want to process multiple channels on one given RippleProcess instance. Real-time constraints dictate that every LFP sample must be processed at Δ*t*_LFP_ < 1/*fs*_LFP_ where Δ*t*_LFP_ is the LFP processing time and *fs*_LFP_ is the LFP sampling rate.

A RippleProcess instance is responsible for processing LFP by filtering the data into the ripple band, estimating the ripple power (or a proxy of it), and determining the start and end times of a ripple. Figure 3*b* shows the computational latency caused by processing LFP data, for a single RippleProcess instance. Here the LFP sampling rate was 1,500 Hz, so the maximum LFP processing latency is 667 μs. These results demonstrate that 10 (and likely more) LFP channels may be processed with comfortable margin to meet the 667 μs real-time deadline. Recall that each Process instance, with the exception of GuiProcess, implements a polling while loop so this deadline represents the upper bound since other tasks must be executed for every loop iteration.

Overall Figure 3 demonstrates favorable scaling characteristics for higher dimensionality data.

## Results

To demonstrate the utility of RealtimeDecoder, we applied it to an experiment to study internally driven memory retrieval. Specifically, we used RealtimeDecoder to deliver reward based on decoded spatial representation to test whether rats can control the activation of specific remote spatial representations . This endeavor is a type of experiment that can decode neural activity while it is occurring and react on a timescale of tens of milliseconds, bringing researchers to a closer understanding of memory-related neural firing patterns.

In this experiment, a Long-Evans rat was surgically implanted with a microdrive with 64 individually adjustable tetrodes (all animal surgery and care was performed in accordance with UCSF IACUC guidelines). Over the course of several weeks, tetrodes were lowered into the CA1 region of dorsal hippocampus. The rat was food-restricted and trained to run on a two-arm maze where it collected sweetened milk reward from lick ports in the maze.

Once tetrodes were in place, the experiment began. Each day consisted of interleaved RUN and REST sessions in the following manner: REST 1, RUN 1, REST 2, RUN 2, REST 3, RUN 3, and REST 4. During REST sessions, the animal was placed in an enclosed box away from the maze.

During RUN sessions, the 2D position of the animal was tracked by Trodes, using the red and green LED arrays attached to the headstage. The 2D position was linearized according to user-defined line segments consisting of 2 approximately equal-length line segments representing the central box and 1 line segment representing each arm, for a total of 4 line segments. For each tracked 2D position, Trodes determined the nearest line segment and output a value between 0 and 1. This value represented the normalized position along the given line segment. RealtimeDecoder was configured with information about the line segments, specifically how many 5 cm position bins were spanned by each line segment. In this way, the system could map the normalized positions output by Trodes to the appropriate position bin used by the decoder. Thus, one position bin in the decoder’s posterior probability space represented 5 cm in the physical world.

**Figure 4.**
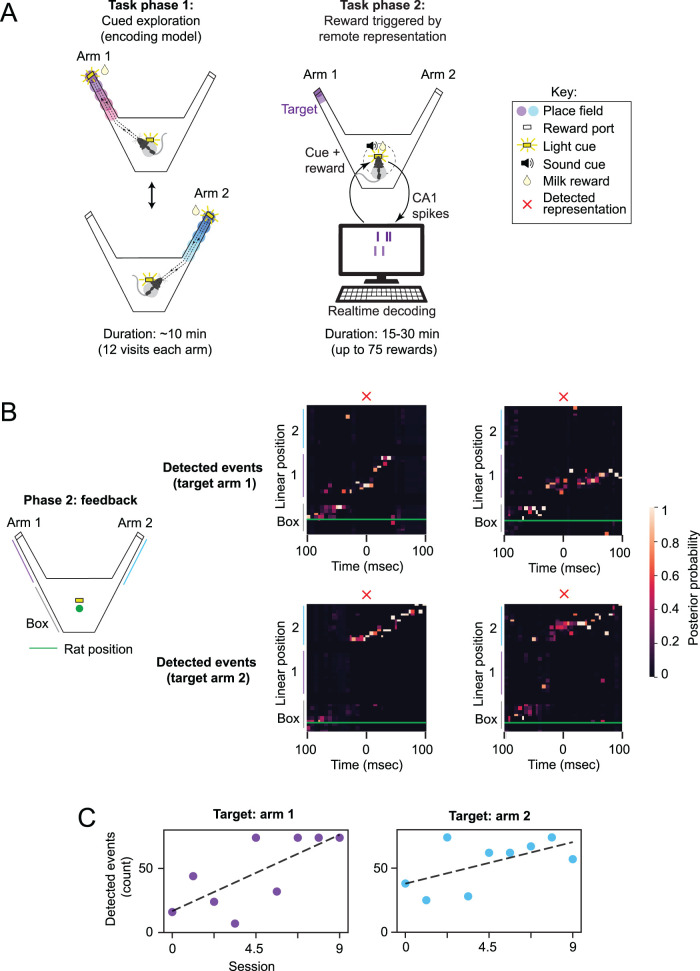
***a***, Overview of experiment to which the real-time decoder was applied. Each session consists of two tasks. In task 1 an animal explores the maze arms. This data is used to train the encoding models. In task 2 the animal receives reward for generating a remote representation event. Although place cells and remote sequences are expected to be present, they are not used directly by the clusterless decoder. ***b***, Example events captured by the decoder. The blue trace is the actual position of the animal. A 100 ms window is drawn around the time of event detection. ***c***, Number of rewards for each session of neurofeedback.

The structure of a RUN session was sub-divided into two tasks (Fig. 4*a*), but the decoder itself runs for the entire duration of a session. During task 1 (∼10 min), a light cue directed the rat to explore each arm (12 visits each). The data collected from this exploration period, and specifically spikes that occurred during periods of running, defined the encoding models contained in the decoder.

In task 2, the animal no longer collected reward by running to the end of a maze arm. Instead, reward was only available at a reward port at the center of the maze, located away from each arm. When the decoder detected a remote representation of a target location unknown to the rat (the end of one arm), a sound cue was played and the rat had 3 s to nose-poke and collect the milk reward. Importantly, remote representations were only rewarded if the rat was near the center port, away from the arms. Detected representations were required to be high-fidelity events i.e., with little representation in the off-target arm.

To detect a remote representation, the most recently decoded activity was used. Specifically every time an update to the posterior distribution occurred, we looked at the most recent 36 ms of decoded activity (i.e., *t*_event_ = 36). The average posterior distribution was computed over this time window, and the result was normalized to sum to 1. Taking this average distribution, we examined the representation in target and off-target regions. If greater than 40% of the distribution was in the target region, <20% was in the off-target region, and the animal was within 17 cm of the central box, then a remote representation was considered to have occurred. In other words, the 40% and 20% thresholds were chosen as indicators of fidelity. Note that we did not include a requirement for a specific LFP signature, although there would likely be some overlap among the events detected by this method and those that detect SWRs or population burst events. Some example events are shown in Figure 4*b*.

One pilot animal performed this task and demonstrated that the closed-loop neurofeedback worked as designed to detect and reward sustained remote representations (Fig. 4*b*). For each session, a minimum of 40 tetrodes were used for decoding. After several days of preliminary neurofeedback, we introduced the requirement that at least 2 tetrodes had spatially specific activity for event detection, so as to reward population-level representations. With this requirement, we found that detected event count increased across 9 feedback sessions targeting arm 1 and then after switching the target region to arm 2, also increased across 9 more sessions (Fig. 4*c*). This provides preliminary evidence that remote hippocampal content can be enriched via reward-based neurofeedback. We confirmed this evidence in subsequent experiments (Coulter et al., 2025).

Overall the following parameters were used: *σ* = 20, Δ*t*_delay_ = 30 ms, Δ*t* = 6 ms, Δ*t*_event_ = 36 ms, two detection thresholds of 40% and 20%, and a total of 41 position bins to represent 205 cm in the physical world (5 cm per bin). The decoder used a uniform non-informative transition model.

## Discussion

Decoding neural activity allows experimenters to assess the structure of ongoing neural representations. Here we present a real-time system that implements a clusterless state space decoder. The specific implementation was designed to decode spatial representations from the spiking of hippocampal neurons, but the system would be relatively easy to modify to decode other variables. We improved upon previous real-time clusterless decoders by writing the software in pure Python to ease the experience of users intending to extend and customize the system for their needs. Despite the use of Python, our system achieved comparable performance to previous systems written with a compiled language, demonstrating the viability of using an interpreted language for this specific application. We also demonstrate the system in a pilot experiment, illustrating how it can be used to detect the expression of specific hippocampal representations of space and to trigger experimental stimuli based on that expression.

One area for improvement is the scalability of our decoder. For best results, one hardware thread is needed for every electrode group. However, the channel counts of neural recording devices are continuing to increase, and even with systems that have many computing cores, the number of channels per device will exceed current central processing unit (CPU) capabilities. One potential solution would be to leverage computing on graphics processing units (GPUs) so that a single hardware thread can support multiple electrode groups. In order to fully take advantage of the GPU, this strategy would likely require some non-trivial programming to convert the synchronous computing into asynchronous. Alternatively, it may be possible to develop dimensionality reduction approaches that make it possible to carry out real-time clusterless decoding from very high channel count probes, something that is currently only possible offline (Zhang et al., 2024).

While these improvements can be made, the current RealtimeDecoder has numerous applications within the neuroscience sub-field of hippocampal physiology by assisting researchers to run many kinds of closed-loop experiments. Applications to other neuroscience sub-fields are also possible. For example one may wish to design a brain–computer interface which can decode memories its human operator is attempting to retrieve, and to subsequently react in real-time based on that decoding. The computational approach illustrated by RealtimeDecoder has the potential to inform solutions to other decoding-based problems in general. Overall, we believe our work serves as a valuable tool to accelerate neuroscience research.

## Appendix

### Customization

Although we have endeavored to write a generalized software system for real-time decoding, it is difficult to anticipate every use case. In some scenarios customization may be necessary to fulfill the particular needs of the experiment. Here we cover different layers of customization and explain the source code at a deeper level so that the user can better understand the modifications that must be made for their specific application.

#### Customizing the data acquisition interface

As explained previously, our software can be likened to a client in a client-server architecture, where RealtimeDecoder is the client and the data acquisition system is the server which sends preprocessed data for decoding. The decoder will work out of the box with Trodes software; for other systems, the user will need to develop a DataSourceReceiver subclass to interface with their data acquisition. The data acquisition is not constrained to be one that streams live data, as in the case of Trodes. One may wish to feed pre-recorded data into RealtimeDecoder, by reading from file. The parent class is defined in Box 3, and an explanation of the intended uses of each method follows.

Box 3. Definition of DataSourceReceiver class

class DataSourceReceiver(MPIClass, metaclass=ABCMeta)

  def __init__(self, comm, rank, config, datatype: datatypes.Datatypes):

    super().__init__(comm, rank, config)

    self.datatype = datatype

  @abstractmethod

  def register_datatype_channel(self, channel):

    pass

  @abstractmethod

  def activate(self):

    pass

  @abstractmethod

  def deactivate(self):

    pass

  @abstractmethod

  def stop_iterator(self):

    pass

  @abstractmethod

  def __next__(self):

    pass

In the __init__() method comm is the MPI communicator, rank is the MPI rank, and config is the dictionary obtained from parsing the configuration file. Finally datatype is one of the datatypes enumerated in the datatypes module.

The register_datatype_channel() method is used to add an electrode group that the object should receive from. Each electrode group may consist of multiple electrodes; for example a tetrode consists of four electrodes.

The activate() method signifies that the DataSourceReceiver is ready to receive data; thus __next__() may return a non-None object.

The deactivate() method signifies that the object is no longer receiving data. In this state __next__() should always return None.

The stop_iterator() stops the object from running entirely. Since the object can be used as an iterator (hence the __next__() method), this method should at the minimum contain a raise StopIteration statement.

Lastly, __next__() should be called whenever data is to be returned. If no data are currently available or the object is in a deactivated state, then this method should return None.

Upon a call to __next__(), any DataSourceReceiver object that returns a data point in the following formats are automatically compatible with RealtimeDecoder. These are further explained below.

Box 4. Definition of SpikePoint class

class SpikePoint(messages.PrintableClass):

  """Object describing a single spike event"""

  def __init__(self, timestamp, elec_grp_id, data, t_send_data, t_recv_data):

    self.timestamp = timestamp

    self.elec_grp_id = elec_grp_id

    self.data = data

    self.t_send_data = t_send_data

    self.t_recv_data = t_recv_data

Spike data points are implemented as in Box 4. Timestamp is a value that marks the timestamp of the data point and can be represented as a 32-bit unsigned integer. elec_grp_id is an integer denoting the electrode group the data point is coming from. data is the actual spike waveform data, a 2D array of shape (nch, nt) where nch is the number of electrodes in the electrode group and nt is the number of time samples in the spike waveform. t_send_data is a 64-bit integer marking the time in nanoseconds where the data was sent out by the data acquisition system. Similarly, t_recv_data is a 64-bit integer marking the time in nanoseconds where the data was actually received by the DataSourceReceiver. Both t_send_data and t_recv_data are primarily used for diagnostics purposes to characterize network latency.

Box 5. Definition of LFPPoint class

class LFPPoint(messages.PrintableClass):

  """Object describing a single LFP data sample"""

  def __init__(self, timestamp, elec_grp_ids, data, t_send_data, t_recv_data):

    self.timestamp = timestamp

    self.elec_grp_ids = elec_grp_ids

    self.data = data

    self.t_send_data = t_send_data

    self.t_recv_data = t_recv_data

LFP data points are implemented as in Box 5. For an LFPPoint timestamp, t_send_data, and t_recv_data have identical meanings as for a SpikePoint. Differences include elec_grp_ids: since a LFPPoint typically may contain data from *multiple* electrode groups elec_grp_ids is a list of those groups. Finally, data is a 1D array containing the actual LFP data.

Lastly position data points are implemented as in Box 6. Both timestamp and t_recv_data have identical meanings to those in SpikePoint and LFPPoint. In a real experiment, the position is often coming from a head-tracked animal, so we will refer to that when explaining the rest of the data that this object represents. segment refers to a line segment (integer-valued) that the animal is closest to. position is a value in [0, 1] that denotes the position along the line segment. 0 and 1 are the ends of the segment. x and y are the x and y position of the animal, respectively, in pixels. x2 and y2 have the same interpretation as x and y, except that this is another point on the animal that is being tracked. For example, the coordinates (x, y) and (x2, y2) may be the position of a red and green LED.

Box 6. Definition of CameraModulePoint class

class CameraModulePoint(messages.PrintableClass):

  """Object describing a single data sample coming from a camera"""

  def __init__(

    self, timestamp, segment, position,

    x, y, x2, y2, t_recv_data

  ):

    self.timestamp = timestamp

    self.segment = segment

    self.position = position

    self.x = x

    self.y = y

    self.x2 = x2

    self.y2 = y2

    self.t_recv_data = t_recv_data

The fastest way to make RealtimeDecoder compatible with a custom data acquisition system is to develop a DataSourceReceiver subclass that still returns a SpikePoint, LFPPoint, or CameraModulePoint. If the experimenter desires to use a different data format, additional development will be necessary so that the software can handle the custom data objects. The most relevant modifications to make will be the next_iter() methods for the RippleManager, EncoderManager, and DecoderManager objects in ripple_process.py, encoder_process.py, and decoder_process.py, respectively.

#### Customizing the record format

When RealtimeDecoder is running, it records results to disk. These can simply be thought of as log entries in binary form. For example, a record is written when the posterior distribution is updated one time step.

Occasionally a user may wish to add, remove, or otherwise change the type of records that are written. At the minimum the appropriate *Manager object should be modified in its constructor. Box 7 shows an example for the DecoderManager.

Box 7. Defining record types

rec_ids=[

  binary_record.RecordIDs.DECODER_OUTPUT,

  binary_record.RecordIDs.LIKELIHOOD_OUTPUT,

  binary_record.RecordIDs.DECODER_MISSED_SPIKES,

  binary_record.RecordIDs.OCCUPANCY

],

rec_labels=[

  ['bin_timestamp_l', 'bin_timestamp_r', 'velocity', 'mapped_pos',

  'raw_x', 'raw_y', 'raw_x2', 'raw_y2', 'x', 'y',

  'spike_count', 'task_state', 'cred_int_post', 'cred_int_lk',

  'dec_rank', 'dropped_spikes', 'duplicated_spikes', 'vel_thresh',

  'frozen_model'] +

  pos_labels + state_labels,

  ['bin_timestamp_l', 'bin_timestamp_r', 'mapped_pos', 'spike_count', 'dec_rank',

   'vel_thresh', 'frozen_model'] +

  likelihood_labels,

   ['timestamp', 'elec_grp_id', 'real_bin', 'late_bin'],

  ['timestamp', 'raw_x', 'raw_y', 'raw_x2', 'raw_y2', 'x', 'y',

   'segment', 'pos_on_seg', 'mapped_pos', 'velocity', 'dec_rank',

   'vel_thresh', 'frozen_model'] +

   occupancy_labels

],

rec_formats=[

  'qqddddddddqqqqqqqd?' + 'd'*len(pos_labels) + 'd'*len(state_labels),

  'qqdqqd?' + 'd'*len(likelihood_labels),

  'qiii',

  'qddddddqdddqd?' + 'd'*len(occupancy_labels)

]

rec_ids are a list of integer-valued numbers, each of which describes the type of record that is being written. These are particularly useful when the outputs of RealtimeDecoder are merged into a single file. Different processes can write the same type of records, so when merging records, the ID is used to group them together.

rec_labels are used to label each element in a single record (i.e., a binary blob). This is useful for converting the binary data into human-readable format, such as a pandas dataframe.

Finally, rec_formats describe the datatype (such integer, float, etc.) used to represent a given record. These are all format strings from the struct module in the Python standard library.

If a record is changed, the corresponding call to write_record() must likewise be updated so that the correct arguments are supplied. Other modifications to the code may be necessary. Since it is impossible to anticipate every possibility, such changes are not described here. Nevertheless this section should point the user in the right direction on where to begin their modifications.

**Extended Data 1.** This file contains the python scripts necessary to run the RealtimeDecoder software. These scripts run all processes described above. Also included in this zip file are configuration files used to customize the software for each application. The most up-to-date version of the code can be found at: https://github.com/LorenFrankLab/realtime_decoder.

**Extended Data 2.** This file contains the python scripts to run the RealtimeDecoder software as well as sample raw data files (neural data and camera data) that allow the user to demonstrate the functionality of the software.

## Synthesis

Reviewing Editor: Michaël Zugaro, CNRS, Collège de France, Inserm Decisions are customarily a result of the Reviewing Editor and the peer reviewers coming together and discussing their recommendations until a consensus is reached. When revisions are invited, a fact-based synthesis statement explaining their decision and outlining what is needed to prepare a revision will be listed below. The following reviewer(s) agreed to reveal their identity: Fabian Kloosterman, Jozsef Csicsvari.

In this manuscript, the authors present the software tool "RealtimeDecoder" for online (real-time) neural decoding that implements a "clusterless" decoding algorithm to detect neuronal population patterns that smoothly transition along a linear scale - e.g., hippocampal place fields on linear tracks. Characterization of the computational latencies showed similar performance as existing solutions, with a short latency of <2ms. The main appeal of the new tool is that it was implemented in Python (as opposed to a compiled language like C or C++), which may make it more convenient for other users to adapt and extend to meet their experimental needs. The authors provided experimental data as well that demonstrate the viability of the methods. Overall, RealtimeDecoder could be a promising addition to the available tools for real-time neural decoding and provide experimenters with extra options to study the relationship between neural activity patterns and cognitive processes. However, we list below concerns regarding some of the claims and suggestions to improve the manuscript.

Major comments

1. The authors claim that their system, contrary to other solutions, is independent of the data acquisition system. In the Appendix, the authors note that their software will work "out of the box" with the Trodes data acquisition software. However, support for other data acquisition systems requires the implementation of new interfaces to those systems. RealtimeDecoder software does not seem to differ from existing solutions (e.g., Falcon) in this respect, as these solutions can also be extended to support other data acquisition systems by adding dedicated classes or plugins.

2. As pointed out by the authors, an implementation in Python (rather than a compiled language like C or C++) may make it easier for users to extend and customize the system for their experiments. However, how easy that will be in practice depends on the complexity of the software and whether its architecture is sufficiently modular and decoupled. Interestingly, the authors added an appendix with a general description of possible ways to extend RealtimeDecoder. Still, it appears that adding support for, e.g., a very different data acquisition system than Trodes (e.g., one that transmits a single raw data stream) may not be trivial. It would be helpful to provide concrete examples (or even implementations) of how other commonly used data acquisition systems could be integrated into RealtimeDecoder.

3. Related to point 2: the software could not be tested, because of its dependency on the Trodes data acquisition system. It would have been useful to include a mode in which data is read from a test data file.

4. The authors write (lines 317-318) that "the system would be relatively easy to modify to decode other variables". It would be more convincing to describe or show examples of modifications (e.g. 2D position decoding, position plus run direction) and discuss the potential impact on model training and latency (e.g., for 2D position decoding, longer training periods and more spikes are likely required, which will slow down the computations).

5. The authors quantify four components of the overall latency for decoding and detection of events but give no estimate of the full round-trip latency from the data acquisition to feedback. It is important to have insight into how the system performs as a whole, which could be assessed for example by feeding a digital pulse back into the acquisition system for each detection and comparing the timing of the pulse to the signal that triggered the detection.

6. Related to point 3: the Trodes data acquisition system appears to perform some preprocessing on the data before they are sent to RealTimeDecoder. What latency is added in Trodes?

7. More details are needed about how exactly the latencies were measured. Was this done during experimental runs, or as separate tests outside of RealtimeDecoder? What were the specifications of the workstation used to perform the tests?

8. Figure 2f: The legend does not accurately describe what is visualized. The plot appears to show the estimation latency during an experiment in which the encoding model is fixed at some point, and no new spikes are added. Thus, the x-axis does not represent the number of spikes in the encoding model (as the legend states), but the number of processed spikes. The point at which the encoding model is fixed should be indicated in the plot.

9. Figure 2e: Does the distribution of p(x,m) estimation latencies include the training period when spikes are still added to the model? If so, that should be clarified in the legend. Preferably, however, the distribution should reflect the estimation latencies for a fixed number of spikes in the encoding model (or separate training from the rest).

10. The descriptions of the event detection latency (in the Methods) and the method for event detection (in the Results) could use clarifications and improvements - as is, they are hard to follow. It would also help if the event detection approach is described in the Methods together with the detection latency.

11. Related to point 8: it is unclear what "The average for each bin (more exactly, position bin) in the distribution is taken across the window." (lines 294-295) means. Also, what threshold (line 295) was used?

12. It would be helpful to have more details on how the tracked position was processed and mapped onto the track. Were positions in the central box also mapped to a 1D line segment?

13. For real-time systems generally a trade-off is made between the speed and accuracy of computations. The authors should assess the decoding and event detection accuracies, for example, by comparing them to an offline approach.

14. For many of the relevant parameters for decoding and event detection, one cannot easily find the specific values that were used to conduct the tests that the authors describe in the Results. The Methods mainly describe the general approach. A section should be added that specifically defines the parameters and setup for the tests (e.g., σ (line 90), Δt_delay and Δt (line 187), Δt_event (line 227), the number of position bins, size of the sliding window for event detection (line 290), threshold for detection (line 295), the use of a uniform non-informative prior).

15. The authors claim that the increase of detected target events (Fig 4c) with reward-based neurofeedback could indicate enrichment of remote hippocampal content. Given that the authors only quantified the target events, it leaves open the possibility that there is a general (i.e., not specific to the target) increase of (remote) content accompanying behavioral changes across sessions. The argument for enrichment would be more convincing if the authors could show that the increase is specific to the target content and that the target-content-triggered reward is required for the increase.

16. Lines 311-312: the authors refer to "subsequent experiments", but these data are missing.

Minor comments

1. Line 143: Does "joint mark intensity function" refer to equation (5)? For clarity, it would be good to mention this equation here and use the same term "joint mark intensity function" when presenting equation (5).

2. The description of the decoding model ("Model formulation") could be improved. In particular:

2a. Make sure all terms are clearly defined (e.g., t_k and Δ_k).

2b. More clearly describe the equations in words, rather than relying on the equations alone. For example, line 71 and equation (2): "prior distribution that incorporates a behavioral model according to a transition distribution ..."

2c. Explain why the likelihood has the form expressed in equation (3) and mention the underlying assumptions.

2d. Line 77: explain the assumption in words

2e. Line 84: the use of the subscripts k and k,j in the pi(...) term is confusing to me - shouldn't it be written without the subscript(s) in the same way as in line 87 to indicate that it "represents a joint probability distribution", rather than the evaluation at specific x_k and m_k,j?

2f. Line 91: should m in p(x,m) have subscript l to indicate that the distribution is evaluated for a specific observed mark m_l?

3. The EncoderProcess seems to take in single spike events and produce an estimate of the joint mark intensity function for each spike at its output. The DecoderProcess, on the other hand, aggregates multiple spike events into time bins and produces a single posterior estimate for each time bin. It would be useful to indicate this in Figure 1. Given that spike data points form a non-continuous data stream, what clock is the DecoderProcess using to aggregate spike data points into time bins?

4. Figure 1: why are there exactly two DecoderProcesses?

5. Figure 1: the output of the DecoderProcess (p(x,I|O)) uses a different notation than in the text.

6. Do each of the Processes in Figure 1 implement buffers on their inputs (or outputs)? Or are points processed as soon as they come in, and new data points that arrive during the processing of a previous data point are dropped? If data points may be dropped, have the authors quantified how often this happens?

7. Line 187: define Δt_delay at first use.

8. Figure 2c: the reason for using a delay would be more clearly visualized if the "usable" spike s3 falls outside the window.

9. Lines 306-305: "After several days of preliminary neurofeedback, we introduced the requirement that at least 2 tetrodes had spatially specific activity for event detection, so as to reward population-level representations." Please explain. Does this mean that the authors were performing the decoding with data from as few as 1 (initial days) or 2 tetrodes? Please specify the number of tetrodes that were used for the tests.

10. Lines 362-363: there is no "previous chapter".
